# Patient Journeys and Outcomes in Mental Healtcare Systems in Europe – Narrative Review and Study Protocol for a Trans-European Comparison

**DOI:** 10.1192/j.eurpsy.2025.692

**Published:** 2025-08-26

**Authors:** H. F. Wiegand, O. Tüscher, K. Adorjan

**Affiliations:** 1 Department of Psychiatry, Psychotherapy and Psychosomatics, University Medicine Halle, Halle (Saale); 2 Department of Psychiatry and PSychotherapy, University Medicine Mainz, Mainz, Germany; 3 Department of Psychiatry and PSychotherapy, University of Bern, Bern, Switzerland; 4 Department of Psychiatry and PSychotherapy, LMU Munich, Munich, Germany

## Abstract

**Introduction:**

Mental healthcare systems in Europe differ greatly in terms of access and care settings. Whereas comparisons of the structures partly exist (e.g. REFINEMENT project), not much is known neither about the patient journeys for specific disease entities nor about outcomes of these differently organized systems.

**Objectives:**

The first objective is to review the existing literature on structural differences in mental healthcare sytsems in Europe. The second objective is to discuss options for studies that gather differences in patient journeys and outcomes for specific disease entities.

**Methods:**

Narrative review about structural differences in mental healthcare in Europe and discussion of a study protocol for assessing patient journeys for 6 important disease entities (Severe Major Depressive Disorder, Schizophrenia, Behavioural disorders in Dementia, Borderline Personality Disorder, PTSD, Alcohol Addiction).

**Results:**

European mental healthcare systems differ greatly in terms of structures of mental healthcare provision. E.g. Belgium had 141 hospital beds per 100,000 inhabitants, Germany 131, the Netherlands 112, Switzerland 95, Austria 75, and Denmark 5 (eurostat, 2024a). However, what these numbers mean for the patient journeys and for the systems outcomes is unclear. We discuss an approach for examining prototypical patient journeys for the above-mentioned disorders and for comparing their outcomes in terms of quality of life and global psychosocial functioning.

**Conclusions:**

We are looking for European partner institutions for establishing a mental health systems research network. The goal is to set up studies that allow to compare patient journeys and outcomes within the differing European Mental Healthcare Systems as a basis for mutual learning from best practice examples.

**Disclosure of Interest:**

None Declared

